# Ventilator-Associated Pneumonia (VAP) in Neurocritical Patients: The Hidden Dialog of Brain and Infection

**DOI:** 10.3390/biomedicines13123112

**Published:** 2025-12-17

**Authors:** Alejandro Rodríguez, Laura Claverias, Ignacio Martín-Loeches, Frederic Gómez Bertomeu, Ester Picó Plana, Sara Rosich, Vanessa Blázquez, Dennis H. Céspedes Torrez, Ruth Lau, María Bodí

**Affiliations:** 1 Critical Care Department, Hospital Universitari de Tarragona Joan XXIII, Mallafre Guasch 4, 43007 Tarragona, Spain; lauraclaverias@gmail.com (L.C.);; 2 IISPV (Instituto de Investigación Sanitaria Pere Virgili), 43005 Tarragona, Spain; 3 Centre for Biomedical Research Network Respiratory Diseases (CIBERES), 43005 Tarragona, Spain; 4 Faculty of Medicine and Health Sciences, Department of Pharmacology, Rovira & Virgili University, 43201 Reus, Spain; 5 Critical Care Unit, Hospital Viamed Tarragona, 43006 Tarragona, Spain; 6 Department of Intensive Care Medicine, Multidisciplinary Intensive Care Research Organization (MICRO), St James’ Hospital, D08 NHY1 Dublin, Ireland; drmartinloeches@gmail.com; 7 Microbiology/Clinical Analysis Laboratory, Hospital Universitari de Tarragona Joan XXIII, 43005 Tarragona, Spain; 8 Faculty of Medicine and Health Sciences, Department of Medicine and Surgery, Rovira & Virgili University, 43005 Tarragona, Spain; 9 Centre for Biomedical Research in Infectious Diseases Network (CIBERINFEC), 28220 Madrid, Spain; 10 Neurosurgical Department, Hospital Universitari de Tarragona Joan XXIII, Mallafre Guasch 4, 43007 Tarragona, Spain

**Keywords:** ventilator-associated pneumonia, traumatic brain injury, cross-talk axis, stroke, functional immunosuppression, risk factors, prognosis

## Abstract

Patients with multiple traumas, particularly those with traumatic brain injury (TBI), are among the most challenging cases in intensive care medicine. Although early orotracheal intubation and invasive mechanical ventilation (IMV) are essential for airway protection and neurological treatment, they significantly increase the risk of lower respiratory tract infection (LRTI), including ventilator-associated pneumonia (VAP) and ventilator-associated tracheobronchitis (VAT). These complications are particularly prevalent among neurocritical patients due to the distinctive interaction between the brain, lungs and immune system. This narrative review examines the current evidence on the mechanisms underlying the brain–lung–immune axis; the diagnostic challenges in identifying respiratory infections in mechanically ventilated TBI patients; and optimal approaches to empirical or quasi-targeted antimicrobial therapy based on diagnostic algorithms and rapid molecular techniques. Severe TBI induces neurogenic inflammation, autonomic dysregulation, and immunosuppression, thereby increasing susceptibility to pulmonary infections. The ‘triple hit hypothesis’ best explains this cascade: sympathetic hyperactivity (first hit), iatrogenic ventilatory injury (second hit), and intestinal dysbiosis with systemic immune dysregulation (third hit). VAP diagnosis remains challenging due to the lack of universal criteria, the overlap with systemic inflammatory response syndrome, and the low specificity of radiological and clinical signs. VAT may represent an intermediate stage within a continuum of ventilator-associated infection. Recent evidence supports the selective use of nebulized antibiotics for VAT, advocating an individualized, locally adapted empirical approach to VAP treatment. Syndromic molecular panels can accelerate the identification of pathogens, enabling the earlier and more appropriate selection of antimicrobials and improving outcomes while preserving stewardship. Understanding the brain–lung–immune axis and improving diagnostic accuracy are essential to enhancing the treatment of respiratory infections in neurocritical care. Integrating clinical assessment, biomarkers and rapid microbiological testing enables timely, targeted therapy and reduces the misuse of antimicrobials.

## 1. Introduction

Patients with multiple trauma, particularly those with traumatic brain injury (TBI), are usually admitted to intensive care units (ICUs) [1,2]. The severity of injury determines the need for invasive interventions to control organ dysfunction and prevent secondary complications. Early orotracheal intubation (OTI) and invasive mechanical ventilation (IMV) are among the most frequent of these measures [3,4,5]. Although often required, OTI increases the risk of lower respiratory tract infection (LRTI) in critically ill patients. This risk remains high despite the implementation of care bundles designed to prevent it [6,7].

LRTIs, including ventilator-associated pneumonia (VAP) and ventilator-associated tracheobronchitis (VAT), occur more often in patients with TBI, especially when head trauma is associated with chest injury [8,9,10,11,12]. Although overall mortality related to VAP has declined [13], its attributable mortality remains a subject of debate. In neurocritical series, VAP is consistently linked to increased episodes of intracranial hypertension, longer durations of mechanical ventilation, and extended ICU stays, even though it has not been associated with higher overall mortality. Several studies suggest that the development of VAP in patients with TBI may significantly worsen prognosis, as indicated by changes in cerebral tissue oxygen pressure (PtiO_2_) and cerebral perfusion pressure (CPP) [11].

The diagnosis of pulmonary infection in mechanically ventilated patients remains challenging [14,15]. The Centers for Disease Control and Prevention (CDC) has proposed a new framework for the automated and objective classification of ventilator-associated events (VAEs) [16]. However, these definitions are intended for epidemiological surveillance and are not applicable to clinical decision-making. Consequently, diagnosing VAP, particularly in patients with TBI, is complex both diagnostically and therapeutically.

Beyond traditional risk factors for VAP, the intricate relationship between the brain, lungs, and systemic immune system contributes to the higher incidence of respiratory infections in this population. The brain–lung–immune axis comprises multiple, often bidirectional, pathways (“cross-talk”) that are not yet fully elucidated [17,18]. In patients with TBI, treatment objectives focus on minimizing secondary brain injury, controlling intracranial pressure (ICP), and maintaining adequate CPP. These aims may conflict with lung-protective ventilation strategies established for the general critically ill population. Furthermore, bronchial infections such as VAT, often accompanied by fever, may lead to antimicrobial treatment that diverges from current international guideline recommendations [19]. As a result, uncertainty persists regarding optimal strategies for the diagnosis and management of respiratory infections in patients with TBI.

This narrative review aims to address three key clinical questions: (1) Why do patients with TBI have a higher incidence of lower respiratory tract infection, considering the complex interaction between the brain, lungs, and immune system? (2) What diagnostic criteria are most appropriate for identifying LRTI in this population? (3) How can empirical or quasi-targeted antimicrobial therapy be optimally initiated using diagnostic algorithms and rapid microbiological testing?

## 2. Cross-Talk Brain–Lung–Immunity Interrelationship

Patients with severe TBI often present with extracranial complications, with pulmonary issues being the most common and associated with a poorer prognosis. Severe TBI can lead to acute respiratory failure (with or without acute respiratory distress syndrome [ARDS] criteria), and patients with ARDS may develop or experience a worsening of primary neurological complications, which can significantly influence their long-term prognosis [17,18,20,21]. This creates a close bidirectional relationship (or ‘cross-talk’) between the brain and the lungs, whereby acute neurological injury can cause pulmonary complications or acute pulmonary injury (including ventilator-induced lung injury [VILI]) can alter cerebral homeostasis, particularly in patients with TBI, thereby complicating the prognosis. In patients with TBI, factors such as elevated intracranial pressure, neurosurgical procedures like decompressive craniectomy and cerebrospinal fluid diversion used to treat it, along with their potential complications, such as infection, can affect systemic inflammatory and autonomic responses. The lung, characterized by its rich vascular network, is essential for gas exchange and systemic oxygenation. Conversely, the brain is the most oxygen-demanding organ in the human body. This interdependence underlies the bidirectional relationship between pulmonary and cerebral pathology, known as the brain–lung–immunity axis [20,21].

This axis encompasses neuroanatomical, endocrine, and immune pathways integrating the central nervous system (CNS), the autonomic nervous system (ANS), the hypothalamic–pituitary–adrenal (HPA) axis, immune mediators, metabolites, microbiota, and gaseous messengers [17,18].

The brain–lung interaction hypothesis has traditionally been described through a dual-hit model [17], in which the activation of inflammatory mediators and a catecholamine surge initiate a systemic immune response and trigger multiple downstream molecular pathways. Within the lungs, these processes raise hydrostatic vascular pressure, increase capillary permeability, and induce capillary vasoconstriction, ultimately resulting in endothelial dysfunction and inflammatory infiltration (ALI, ARDS).

More recently, this framework has evolved into the triple-hit hypothesis [17,18,22], which offers a more comprehensive understanding of the pathophysiological cascade linking cerebral injury to pulmonary dysfunction. The first hit arises from sympathetic hyperactivity following acute brain injury, driving inflammation, oxidative stress, and enhanced pulmonary vulnerability. The second hit involves secondary iatrogenic insults (most notably mechanical ventilation or lung infection) that amplify this vulnerability through ventilator-induced lung injury mechanisms [22].

**Figure 1:**
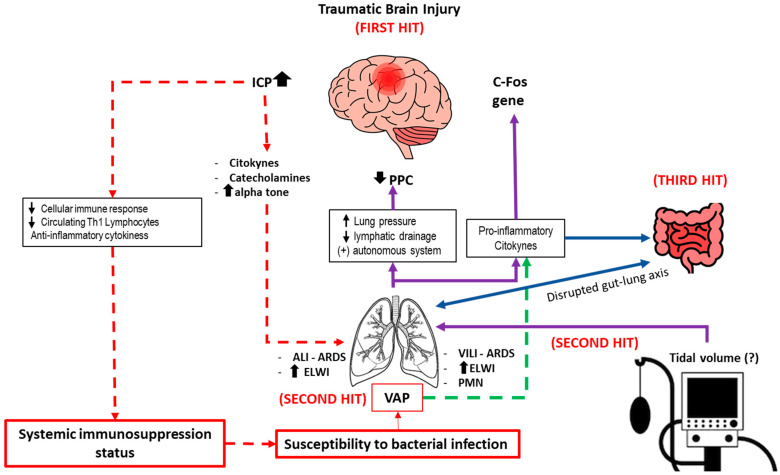
This schematic illustrates the ‘triple hit’ hypothesis. The initial primary brain injury triggers sympathetic hyperactivity and a catecholamine surge, resulting in lung injury (First hit). This early insult contributes to acute respiratory failure (ALI, ARDS) and renders the lungs more vulnerable to subsequent harmful stimuli, including mechanical ventilation (particularly with high tidal volumes) and infection (Second hit). In parallel, dysfunction of the gut–lung axis, characterized by dysbiosis and impaired intestinal integrity, leads to immune dysregulation and microbiome alterations that subsequently influence pulmonary function (Third hit). (Abbreviations: TBI: Traumatic brain injury; VAP: ventilator-associated pneumonia; ALI: acute lung injury; ICP: intracranial pressure; ARDS: Acute respiratory distress syndrome; ELWI: Extravascular Lung Water Index; VILI: Ventilator-induced lung injury; PMN: polymorphonuclear cells).

The third hit integrates the effects of gut dysbiosis and intestinal barrier dysfunction, which provoke systemic immune dysregulation and microbiome alterations that extend their deleterious impact to lung tissue. Together, these sequential events form a neuro-immune-microbial continuum in which primary cerebral injury propagates multi-organ dysfunction through interconnected autonomic, inflammatory, and microbial pathways. The triple-hit model thereby refines current conceptualizations of neurogenic pulmonary injury, underscoring the interplay between sympathetic over-activation, therapeutic interventions, and gut-lung crosstalk as key drivers of respiratory morbidity following acute brain insults (Figure 1).

### 2.1. The Autonomic Component

The autonomic component is primarily mediated by the vagus nerve (parasympathetic) and upper thoracic sympathetic fibers. Vagal stimulation promotes bronchoconstriction, mucus secretion, and mucosal edema, whereas sympathetic activation produces bronchodilatation and reduces vascular permeability. Importantly, the vagus nerve provides both efferent and afferent communication between the lung and CNS, forming the neural substrate of the brain–lung axis [7,23].

Following traumatic brain injury or stroke, vagal dysfunction can impair swallowing and cough reflexes, increasing the risk of aspiration and pulmonary infection. Moreover, the vagus nerve modulates inflammation via the cholinergic anti-inflammatory pathway (CAP) [7]. Afferent signals from inflammatory cytokines activate the nucleus tractus solitarius, while efferent acetylcholine release acts on α7 nicotinic acetylcholine receptors (α7nAChR) on macrophages to suppress pro-inflammatory cytokine production. Loss of vagal tone or α7nAChR deficiency exacerbates pulmonary inflammation and infection. However, excessive CAP activation may induce immunosuppression, heightening susceptibility to pneumonia or stroke-associated pneumonia (SAP) [7,23,24], illustrating its dual role in pulmonary immunity (Figure 1).

Additionally, pulmonary neuroendocrine cells (PNECs) serve as specialized airway sensors mediating neuroimmune responses. These cells secrete neuropeptides such as substance P, bombesin, and calcitonin gene-related peptide, which regulate vascular tone, inflammatory cell recruitment, and epithelial function. Substance P, in particular, links nociceptive vagal activity after brain injury to pulmonary inflammation and vascular permeability [7,23,24].

Finally, the sympathetic nervous system (SNS), through catecholamine release from the sympathetic-adrenomedullary system, contributes to stress-related pulmonary responses.

### 2.2. The Endocrine Component

The endocrine component of the brain–lung–immunity axis is primarily governed by the hypothalamic–pituitary–adrenal (HPA) axis and the Sympathetic-Adrenomedullary System (SAS), which together orchestrate neuroendocrine responses to stress and maintain pulmonary homeostasis [7].

The HPA axis, comprising the hypothalamus, pituitary, and adrenal glands, operates through a feedback circuit mediated by corticotropin-releasing hormone (CRH), adrenocorticotropic hormone (ACTH), and glucocorticoids (GCs). Acute activation of this axis facilitates anti-inflammatory and immunosuppressive effects by mobilizing lymphocytes, inhibiting macrophage activity, and suppressing Th1 responses [25]. However, TBI may weaken airway immunity, impair mucociliary clearance, and predispose individuals to respiratory infections and asthma exacerbations. Excess GCs also induce epithelial apoptosis and compromise the integrity of the airway barrier (Figure 1).

The SAS complements HPA activity during acute TBI by releasing catecholamines (epinephrine, norepinephrine, dopamine), which enhance cardiovascular and metabolic adaptation but may also promote infection. Norepinephrine (NE) increases bacterial proliferation and virulence, impairing host defense in the respiratory tract. NE additionally modulates immunity by suppressing Th1 cell activation and altering cytokine balance via β-adrenergic signaling, contributing to post-stroke immunosuppression and infection risk [7,25].

Overall, the integration of the HPA axis and SAS within the brain–lung axis underscores the tight neuroendocrine control of respiratory immunity and stress adaptation. While moderate activation maintains pulmonary defense and homeostasis, acute or excessive stimulation promotes airway inflammation and infection, highlighting endocrine pathways as potential therapeutic targets in respiratory disease.

### 2.3. The Immune Component

The immune component forms a pivotal component of the brain-lung-immune axis, mediating bidirectional communication between the central nervous system (CNS) and the respiratory system. This interaction integrates immune organs, cells, and signaling molecules to maintain systemic homeostasis while enabling mutual modulation between neural and pulmonary processes [7].

Following TBI, intracerebral hemorrhage or ischemic stroke, inflammatory cytokines and damage-associated molecular patterns (DAMPs) cross the blood–brain barrier (BBB), triggering systemic immune activation. These circulating mediators promote neutrophil infiltration, alveolar damage, and secondary lung injury. Moreover, signaling pathways such as HMGB1–RAGE contribute to post-stroke immunosuppression and heightened susceptibility to stroke-associated pneumonia [7,26].

The CNS also regulates pulmonary immunity via autonomic and endocrine mechanisms. The CAP, mediated by α7nAChR on macrophages, suppresses pro-inflammatory cytokine production. Similarly, glucocorticoids and catecholamines, released through activation of the HPA axis and SAS, dampen Th1 responses, inhibit macrophage activity, and modulate cytokine profiles, collectively predisposing to pulmonary infection under chronic stress conditions [7].

Conversely, pulmonary inflammation can detrimentally affect the CNS. Circulating cytokines (e.g., TNF-α, MMP-9) generated during acute lung injury (ALI) or chronic infection penetrate the CNS, activating glial cells and promoting neuronal death. Excessive immune activation can lead to severe neuroinflammation. The release of catecholamines by the lungs leads to the activation of the c-Fos gene in the central amygdala, the hippocampus, the hypothalamic paraventricular nucleus and the supraoptic nucleus. When tidal volume (Vt) is elevated, c-fos activation is also observed in the retrosplenial cortex. The c-fos gene is a marker of neuronal activation and correlates well with increases in brain function and metabolism. It is involved in neuronal plasticity, a phenomenon expressed in response to a wide range of stimuli, and implicated in processes such as transcription, apoptosis, and proliferation. Conversely, the release of pro-inflammatory substances from the lung in cases of acute lung injury (ALI) can affect the brain, as well as alter regional cerebral perfusion due to an increase in mean airway pressure, a decrease in lymphatic drainage and activation of the autonomic nervous system (Figure 1) [7,24,25,26,27].

Recent studies indicate that the lung may act as a reactivation site for autoreactive T cells, which subsequently migrate to the CNS to induce autoimmune diseases such as multiple sclerosis, linking respiratory inflammation to neurological relapse. Alterations in the pulmonary microbiota also modulate microglial reactivity and CNS susceptibility to autoimmunity, underscoring the immunological interdependence between lung and brain.

In summary, this is a critically ill patient with functional immunosuppression secondary to severe traumatic brain injury, reflecting the complex brain–lung–immune cross-talk. The presence of multiple invasive devices to manage secondary complications further amplifies the risk of healthcare-associated infections, most notably ventilator-associated pneumonia, which represents the principal and most serious respiratory complication in this context.

## 3. Incidence of VAP: The Problem with Diagnosis

The diagnosis of VAP continues to represent one of the most complex and debated issues in intensive care medicine. Despite decades of research and the publication of international guidelines, no universally accepted diagnostic standard exists, leading to striking variability in reported incidence and interpretation. Depending on the criteria applied, the incidence of VAP ranges from 4% to 42% among critically ill populations [3,13,28,29]. In neurocritical patients, rates are considerably higher, between 10 and 47 episodes per 1000 ventilator days [3,13], and may exceed 50 per 1000 ventilator days when concomitant polytrauma or thoracic injury is present [28,29]. This disparity largely reflects the limited sensitivity and specificity of current diagnostic tools and definitions, which compel clinicians to navigate the delicate balance between overtreatment with broad-spectrum antibiotics and delayed therapy that risks adverse outcomes. Table 1 shows the differential criteria between VAP and VAT. However, these only reflect an approximation of the suspected diagnosis.

The gold standard for confirming VAP remains histopathological evidence of infection and inflammation within lung parenchyma. However, obtaining such specimens is rarely feasible or appropriate in the critically ill, and post-mortem examinations offer little clinical benefit. Consequently, clinicians depend on a composite diagnosis that combines clinical, radiological, microbiological, and laboratory markers, among which procalcitonin has emerged as a potentially useful biomarker [30,31]. Yet, the classical clinical triad of fever, purulent respiratory secretions, and leukocytosis remains nonspecific. These signs are commonly observed in conditions characterized by systemic inflammation, such as TBI, polytrauma, chronic obstructive pulmonary disease (COPD), or ARDS. Moreover, radiological findings, including new or progressive pulmonary infiltrates, often overlap with atelectasis, fluid overload, or pulmonary edema, further clouding the diagnostic picture.

Within this diagnostic uncertainty, VAT has emerged as an intermediate or parallel entity that further complicates interpretation [32,33,34,35]. VAT shares many of the clinical manifestations of VAP, such as fever, increased tracheal secretions, and positive microbiological cultures, but lacks the radiographic evidence of pneumonia. Although sometimes dismissed as a benign colonization process, evidence suggests that VAT may represent an early or less severe stage along the spectrum of ventilator-associated lower respiratory tract infection, bridging the gap between airway colonization and pneumonia.

In a study by Karvouniaris et al. [32], neurosurgical patients demonstrated a significantly higher frequency of VAT compared with other ICU populations (28.5% vs. 14.1%, *p* = 0.02). While VAT was not associated with increased ICU mortality, it was linked to prolonged mechanical ventilation, extended ICU stay (odds ratio [OR] = 3.04; 95% confidence interval [CI]: 1.35–6.85), and longer hospitalization (OR = 2.25; 95% CI: 1.04–4.9). This suggests that, although VAT may not directly increase the risk of death, it contributes to morbidity, resource utilization, and prolonged exposure to invasive devices, thereby increasing the risk of subsequent infection.

From a pathophysiological perspective, VAT and VAP likely form part of a continuum of infection driven by a dynamic interaction between host defenses, bacterial virulence, and risk exposure, particularly mechanical ventilation [14,32]. In this model, airway colonization represents the initial step, progressing to inflammation and alveolar infection once local immune mechanisms are overwhelmed. However, not all patients traverse this continuum; some clear bacterial colonization spontaneously, while others develop pneumonia without preceding VAT, illustrating the heterogeneity of the host–pathogen response [14].

Distinguishing between these entities remains challenging, as radiographic progression of infiltrates cannot reliably differentiate pneumonia from inflammatory or non-infectious pulmonary processes. Thus, the diagnosis of VAP must be contextual, integrating temporal, clinical, and microbiological information rather than relying on isolated findings. Novel diagnostic strategies, including quantitative cultures, molecular assays, and biomarker-guided algorithms, offer promise but have yet to achieve widespread standardization or validation across neurocritical populations.

The use of procalcitonin (PCT)-guided protocols to initiate antimicrobial treatment has been extensively evaluated across different patient populations [36,37,38]. Recent meta-analyses indicate that PCT-guided strategies are associated with reduced antibiotic consumption, fewer antibiotic-related adverse effects, and lower mortality [36]. Despite these findings, clinical guidelines for VAP [29] do not recommend using PCT to guide the initiation of antimicrobial therapy, thereby leaving the debate unresolved. However, only a limited number of studies have assessed the performance of PCT in neurosurgical ICU (NICU) patients. Neurocritical patients are particularly complex and may exhibit neurogenic fever or other neurological sources of inflammation related to the primary cerebral insult that led to ICU admission. Such inflammatory responses may compromise the diagnostic accuracy of biomarkers used to identify infection. Several studies have examined PCT levels in neurocritical patients for the diagnosis of hospital-acquired pneumonia (HAP) and VAP [39,40,41,42]. Although isolated studies have reported findings that challenge the usefulness of PCT, the majority have identified PCT as a highly specific predictor of sepsis in this population. More recently, one study [39] reported that a PCT cut-off of 0.095 ng/mL demonstrated high diagnostic accuracy for HAP in NICU patients (AUC ROC > 90%) and could support earlier identification and treatment. Nonetheless, reaching firm conclusions remains difficult due to heterogeneity in diagnostic criteria and variability in the populations studied.

From a clinical standpoint, these diagnostic ambiguities have significant implications for antimicrobial stewardship. Overdiagnosis of VAP promotes unnecessary antibiotic use, fostering resistance pressure and secondary infections, whereas underdiagnosis delays essential therapy, worsening outcomes. Therefore, establishing clear diagnostic boundaries between VAP and VAT is not merely academic but fundamental to optimizing patient management, reducing morbidity, and improving ICU efficiency [32,43,44,45].

**Figure 2:**
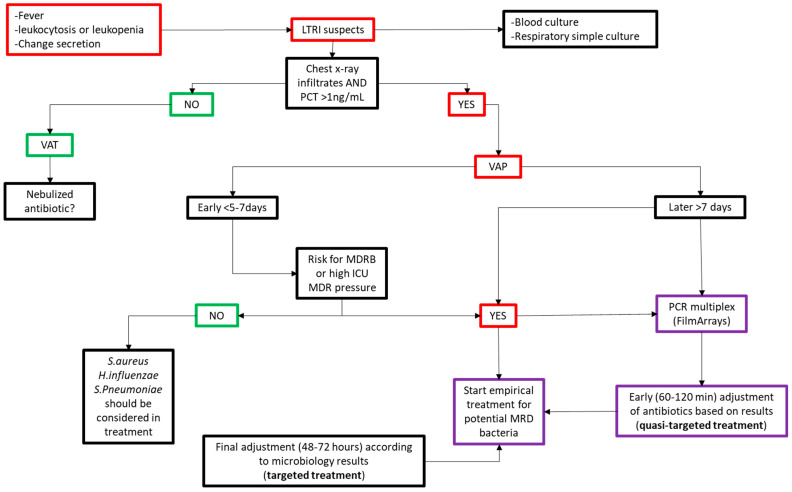
Algorithm for the diagnosis and treatment of ventilator-associated pneumonia (VAP) in neurocritical patients. (Abbreviations: VM: invasive mechanical ventilation; MDRB: multi-drug resistant bacteria; PCR: Polymerase Chain Reaction).

VAP and VAT should not be viewed as discrete, mutually exclusive conditions, but as interconnected stages within a dynamic infection continuum. Accurate differentiation requires a holistic diagnostic approach that integrates clinical acumen, imaging, microbiology, and biomarkers, tailored to the patient’s neurological and systemic context (Figure 2).

## 4. Risk Factors

The pathogenesis of nosocomial respiratory infections is complex, and the numerous risk factors for VAP in ventilated patients are well known. All general risk factors also apply to patients with TBI, in whom immune alterations due to cross-talk between the brain, lung, and immune systems favor the development of these infectious complications [17,18]. Similarly, care bundles designed to reduce the incidence of VAP in ventilated patients also apply to patients with TBI. Table 2 lists the mandatory measures to be taken for ventilated patients in accordance with the Spanish Ministry of Health’s national Zero Pneumonia Plan [46]. Although the Pneumonia Zero care bundle is local, its measures draw on the best available international evidence and are largely consistent with those proposed by other authors [47,48,49,50] (Table 2). Some preventive measures have strong evidence to support their routine use, while others have been shown only to be associated with reductions in the transmission or spread of resistant microorganisms.

Elevating the head of the bed to more than 30° is one of the oldest and simplest measures, since gastro-esophageal reflux worsens in the supine position. It has been argued that a semi-recumbent posture might encourage the gravitational movement of oropharyngeal secretions towards the airways, potentially increasing the risk of aspiration around the cuff of the endotracheal tube. Although study results have at times been contradictory [51,52], current evidence reviews and guideline discussions support maintaining head-of-bed elevation between 30° and 45° as the safest position for preventing ventilator-associated pneumonia in hemodynamically stable patients [47]. Early clinical trials and bundle implementations also pointed to benefits of a semi-recumbent position in lowering VAP risk.

The semi-recumbent position is straightforward, poses minimal risk, and facilitates patient mobility and weaning from mechanical ventilation more effectively than the supine position. For these reasons, it remains a fundamental element of the prevention care bundle.

Reducing sedation and performing daily wake-up tests (spontaneous awakening trial –SAT- or spontaneous breathing trial –SBT-) are standard practices for weaning patients from IMV once their acute condition has stabilized. In patients with TBI, however, the timing of sedation reduction or daily assessments often diverges considerably from that of the initial intubation. Overall, daily wake-up tests have been associated with a 2–4 day reduction in the duration of mechanical ventilation, and this benefit has been extrapolated to a decreased risk of VAP. A recent US study [53] reported that deep sedation or coma, as well as the administration of benzodiazepines or ketamine, were independently associated with a lower likelihood of achieving a SAT or SBT on the following day. In addition, a recent meta-analysis [54] demonstrated that implementing ICU care bundles incorporating SAT can reduce ICU length of stay, time on mechanical ventilation, delirium, and ICU and hospital mortality, while also promoting early mobilization in critically ill patients. Accordingly, SBT and the avoidance of excessive sedation should be considered essential components of all VAP prevention bundles, with the overarching aim of reducing patients’ cumulative exposure to risk.

Subglottic drainage involves the use of specially designed endotracheal tubes that allow oropharyngeal (subglottic) secretions to be aspirated from above the cuff. This reduces the bacterial load that may leak into the lower airways and can help prevent the development of VAP, particularly early-onset VAP. Although several studies [55,56] have produced inconclusive results regarding the effectiveness of subglottic suction tubes, a recent meta-analysis [57] reported that VAP prevention bundles combining adequate endotracheal tube cuff pressure with subglottic suction achieved the most substantial reductions in VAP incidence, lowering rates by more than 50%.

With respect to cuff pressure, multiple studies [58,59] have demonstrated the importance of ensuring appropriate pressure levels. However, more frequent monitoring, or the continuous automatic regulation of cuff pressure using pneumatic devices, has not been shown to be more effective than standard care in preventing VAP [60].

Selective digestive decontamination (SDD) consists of applying an oral paste and administering a suspension containing colistin, tobramycin, and nystatin via a nasogastric tube, maintained throughout the period of invasive mechanical ventilation. In addition, SDD includes a short course of intravenous antibiotics (typically a third-generation cephalosporin) for four days. Several studies [61] have reported that SDD is associated with reductions in ventilator-associated pneumonia (VAP) and mortality in critically ill patients. However, a more recent randomized trial comparing SDD with placebo found no difference in mortality [62].

The principal concern regarding the routine use of SDD and selective oral decontamination (SOD) relates to their potential long-term impact on antimicrobial resistance, particularly concerning third-generation cephalosporins. Oostdijk et al. observed that both rectal and respiratory colonization with ceftazidime-resistant strains doubled after the SDD period, having initially decreased during the SOD phase (from 5% to 15%; *p* < 0.05). It is generally accepted that in settings with a low prevalence of multidrug-resistant organisms, SDD is associated with reduced antibiotic resistance and improved patient outcomes. Conversely, in environments with moderate to high prevalence of antimicrobial resistance, the benefits of SDD on clinically meaningful outcomes have yet to be convincingly demonstrated [47].

Patients with TBI have additional risk factors that differentiate them from critically ill patients in general. Table 3 shows the risk factors for VAP in neurocritical patients, as identified by various authors. Some of these studies examine the factors according to when VAP develops, while others report on their overall importance.

As can be seen, most authors recognize that head trauma, especially when associated with trauma in other locations, is a factor closely related to the development of VAP. Additionally, severe chest trauma (AIS < 3) is associated with a high risk of VAP, potentially due to pulmonary contusions favoring infection [63]. Administering barbiturates to manage very high levels of intracranial pressure that do not respond to usual measures is another factor associated with VAP development, as it causes greater immune system alteration. Gastric aspiration is another observed risk factor. Macroaspiration and microaspiration of gastric contents during the orotracheal intubation process, especially in patients treated with anti-H2, may be strong risk factors for VAP. With regard to this point, and bearing in mind point 8 of the Spanish care bundles, the early administration of antibiotics (within 24 h) after orotracheal intubation is recognized as a protective factor against VAP. Although this is a mandatory measure, its impact is currently controversial.

Administering intravenous antibiotics appears to prevent the development of VAP in this population and may reduce the associated adverse consequences. A previous systematic review addressed the question of whether prophylactic antibiotic use is associated with a reduction in VAP incidence [68]. However, this review included only two randomized clinical trials. The authors concluded that prophylactic antibiotic administration in this population was associated with a reduction in VAP incidence and length of ICU stay, but not with reduced mortality. It should be noted that VAP was not defined uniformly in the included studies, which makes the results difficult to interpret. This is particularly pertinent given that subsequent randomized clinical trials in populations with severe acute brain injuries have produced inconclusive results [69,70,71,72].

In a recent meta-analysis, Hadley-Brown et al. [69] found no clear association between the administration of prophylactic antibiotics and reduced mortality in this population. However, the authors did find an association between prophylactic antibiotic administration and a reduced VAP diagnosis, although the certainty was considered low.

It is worth noting the discrepancy between the finding of reduced VAP and the lack of an associated reduction in mortality. While this discrepancy may be due to the small trials conducted to date lacking the power to detect a difference in mortality, it is more likely that we should not associate an initial measure intended to prevent early VAP, which is more closely related to gastric aspiration during intubation, with ICU patient mortality, which depends on many factors that vary over time. Therefore, crude ICU mortality may not be the ideal outcome measure for evaluating the efficacy of prophylactic antibiotic interventions in patients with acute severe traumatic brain injury [7]. However, diagnostic difficulties create uncertainty about the true impact of this measure, as warning signs (e.g., fever, leukocytosis, and change in secretions) may also be related to the primary pathological characteristics of the brain. Furthermore, the vast majority of studies included in meta-analyses have an extremely high incidence of VAP in the control group (>40–50%), suggesting that an intervention could have a favorable impact on this outcome. However, the impact that this measure could have in units where the NAV incidence is substantially lower has not been demonstrated.

One possible alternative to the prophylactic intravenous administration of antibiotics is the nebulized administration of antibiotics [73]. This innovative treatment method allows antibiotics to be delivered in high concentrations to the tracheobronchial tree where gastric contents have been aspirated. Although a meta-analysis conducted in 2018 observed that preventive nebulized antibiotics can reduce the incidence of VAP, the findings are conflicting, given that most patients received treatment via intratracheal instillation rather than nebulization, which is an obsolete and ineffective method of administration [74]. A recent meta-analysis [73], which included a total of 1160 participants from four trials, found that nebulized antibiotic inhalation was associated with a reduction in the incidence of VAP in mechanically ventilated patients. There was no impact on ICU and hospital mortality, adverse events, duration of mechanical ventilation, or length of stay in the ICU or hospital.

In the era of multidrug resistance, restrictive antibiotic administration policies must closely align with the clear benefits of their use as a prophylactic measure. In ICUs with a high incidence of VAP in neurocritical patients, implementing prophylactic intravenous or nebulized antibiotics (the preferred option) may be the most appropriate way to prevent VAP in patients with TBI.

## 5. Epidemiology and Empirical Treatment Approach

### 5.1. Epidemiology

Orotracheal intubation combined with mechanical ventilation increases the risk of developing bacterial pneumonia by six- to twenty-fold [2,6,14]. The presence of invasive devices such as the endotracheal tube (ETT) and orogastric or nasogastric tubes (OGT/NGT) facilitates bacterial entry into the lower respiratory tract, promoting oropharyngeal colonization at an estimated rate of 1% per day.

The primary mechanism of bacterial inoculation into the lower airways is aspiration, which may occur as macroaspiration (during intubation or as a consequence of post-TBI-related sensory impairment), or as microaspiration (due to leaks around the ETT cuff or passage of contaminated secretions through the ETT lumen). Additionally, bacteria readily form biofilms along the inner surface of the ETT, where they proliferate, persist, and periodically dislodge as biofilm emboli, contributing to lower respiratory tract infection [14].

Although the pathophysiology of VAP and VAT in neurocritical patients parallels that seen in other critically ill populations, several distinctive factors modify their course. The bacterial load, pathogen virulence, and host immune competence are tightly interlinked. In patients with TBI, the brain–lung–immune cross-talk axis plays a critical role: neurogenic immunosuppression, autonomic dysregulation, and impaired airway reflexes collectively diminish host defenses, increasing susceptibility to infection. Whether colonization progresses to overt lower respiratory tract infection depends on this delicate balance between microbial aggression and host vulnerability [17,18].

Traditionally, nosocomial lower respiratory tract infection has been defined as the onset of respiratory infection after 48 h of hospitalization or mechanical ventilation. Within this framework, VAP is classified as either early-onset or late-onset. Early-onset VAP, occurring within the first 5–7 days of mechanical ventilation, typically results from endogenous flora already present in the oropharynx, and is usually caused by antibiotic-sensitive microorganisms. In contrast, late-onset VAP, developing after this period, is often associated with multidrug-resistant (MDR) pathogens, acquired through ICU colonization [75,76,77].

This temporal and microbiological distinction carries major clinical implications. Early VAP is generally associated with a more favorable prognosis, while late-onset VAP entails greater morbidity and mortality due to therapeutic challenges posed by MDR organisms [75,76,77]. The pathogens most frequently isolated in neurocritical patients with VAP are summarized in Table 4.

As demonstrated by the reviewed studies, there is considerable heterogeneity in the microorganisms isolated from VAP in neurocritical patients. This variability may reflect geographical differences, but is more plausibly explained by unit-specific antimicrobial practices and infection control policies. Interestingly, studies from France, Canada, Spain, Italy, and Colombia, despite their diverse settings, consistently identify methicillin-sensitive *Staphylococcus aureus* (MSSA) as the predominant pathogen in this population. In contrast, reports from Serbia, Greece, and South Africa reveal a predominance of potentially MDR organisms, illustrating the global divergence in bacterial ecology.

Notably, the distinction between early- and late-onset VAP does not consistently correlate with differences in microbial profiles, as highlighted by Jovanovic et al. [9], who found no clear temporal association with specific pathogens. This underscores the importance of understanding local epidemiological patterns to guide empirical antibiotic selection accurately and effectively.

Overall, MSSA, *Haemophilus influenzae*, and *Streptococcus pneumoniae* remain the most frequently isolated microorganisms in patients with traumatic brain injury (TBI) who develop VAP/VAT, irrespective of onset timing. However, in ICUs with a high prevalence of MDR organisms, early-onset VAP may already involve pathogens such as *Pseudomonas aeruginosa* and *Acinetobacter baumannii*. In these settings, achieving appropriate empirical antibiotic coverage becomes particularly challenging, and broader-spectrum initial therapy may need to be considered to reduce treatment failure and mortality.

### 5.2. Empirical Treatment Approach

Patients admitted to the intensive care unit (ICU) represent a uniquely complex population, often presenting with multiple pathologies that obscure the source of systemic symptoms and complicate diagnostic interpretation. In mechanically ventilated patients, positive respiratory cultures are common, yet differentiating airway colonization from true infection remains a major challenge. Although new systemic features, such as fever, leukocytosis, or changes in respiratory secretions, may suggest infection, these findings frequently overlap with systemic inflammatory response syndrome (SIRS) or other sources of inflammation that prompted ICU admission. Consequently, the diagnosis of VAP and VAT remains difficult, and therapeutic decisions are often made under conditions of uncertainty [80].

While most studies indicate that VAT is not independently associated with increased mortality, it consistently correlates with prolonged mechanical ventilation, longer ICU stays, and greater healthcare costs. Early and appropriate antimicrobial therapy has been shown to reduce the progression of VAT to VAP [34], yet overdiagnosis and overtreatment carry their own risks, namely adverse drug effects and microbiological resistance pressure. A post hoc analysis from a large multicentre US ICU trial [81] revealed that 60% of patients with SIRS continued to receive antibiotics unnecessarily, largely due to the absence of clear diagnostic criteria. This diagnostic ambiguity mirrors the blurred distinction between SIRS and sepsis, highlighting the urgent need for precise definitions and rational antibiotic use in VAT and VAP.

There is growing consensus that colonization, VAT, and VAP exist along a pathophysiological continuum, and that the key clinical challenge lies not in recognizing infection but in defining its stage and severity. Advocates for VAT treatment argue that withholding or delaying antibiotics may worsen outcomes by allowing progression to VAP, prolonging ventilation and ICU length of stay, and complicating weaning. The first randomized controlled trial (RCT) on VAT treatment demonstrated a significant reduction in ICU mortality among treated patients (18% vs. 47%; OR 0.24, 95% CI 0.07–0.88), and a lower incidence of VAP (13% vs. 47%; OR 0.17, 95% CI 0.04–0.70) [43]. Similarly, observational data in patients with MDR pathogens found that appropriate antibiotic therapy markedly reduced progression to VAP (OR 0.12, 95% CI 0.02–0.59, *p* = 0.009) [44]. However, the universal treatment of VAT would entail a substantial increase in antibiotic use, particularly in units with high prevalence (up to 28.5% in neurosurgical ICUs), with the potential to exacerbate antimicrobial resistance, including difficult-to-treat organisms such as *Pseudomonas aeruginosa* and *Acinetobacter baumannii*, and increase drug-related toxicity. Accordingly, the clinical benefit of treating all VAT cases remains controversial. Determining whether specific subgroups of patients, such as those with severe neurological injury, immunosuppression, or MDR colonization, may benefit most from targeted therapy is a key research priority.

The 2016 Infectious Diseases Society of America (IDSA) guidelines on the management of ventilator-associated pneumonia (VAP) [29] emphasize the importance of appropriate treatment while acknowledging that antibiotic therapy in VAT may shorten the duration of mechanical ventilation, though there is no consistent evidence of benefit in terms of mortality or ICU length of stay. Consequently, the guidelines do not recommend routine antibiotic treatment of VAT, citing wide variability in diagnostic definitions and the limited quality of available clinical trials.

Nonetheless, the IDSA guideline [29] underscores the need for individualized clinical judgment, recommending antibiotic therapy in severe or deteriorating cases where both clinical and microbiological evidence strongly suggest infection. This consideration is particularly relevant for patients with TBI, in whom fever and hypoxemia secondary to VAP can significantly influence cerebral perfusion and potentially contribute to poorer neurological and overall outcomes.

In such cases, we propose an approach (Figure 2) involving targeted management of VAT through the use of nebulized antibiotics as a limited, localized therapy. Empirical treatment of VAP should be adapted to the duration of mechanical ventilation, local epidemiological data, and the prevalence of multidrug-resistant (MDR) pathogens within the unit.

The use of rapid molecular diagnostic techniques, such as syndromic PCR panels, has been shown to markedly reduce the time required to identify the causative pathogen, thereby increasing the likelihood of administering appropriate empirical or quasi-targeted antimicrobial therapy [82,83]. This is particularly valuable in patients with late-onset VAP and in ICUs with a high prevalence of MDR organisms [84]. Such syndromic panels are becoming increasingly available in ICUs due to their substantial impact on antimicrobial consumption and their higher rates of appropriate therapy compared with conventional empirical treatment. Despite the cost of the kits, these improvements render the technique cost-effective. Although syndromic panels are limited by their inability to reliably distinguish colonization from infection and by their failure to detect pathogens not included in the assay, they should nonetheless be considered in patients with a high pre-test probability of respiratory infection, as part of a structured diagnostic and therapeutic protocol embedded within antimicrobial stewardship programs [84].

The diagnostic-therapeutic algorithm we propose (Figure 2) integrates this approach, facilitating the early optimization of empirical therapy as soon as molecular results become available. In settings where these technologies are not accessible, empirical treatment must be closely aligned with local microbiological surveillance data. This underscores the fact that a universal ‘one size fits all’ strategy is neither realistic nor advisable.

In summary, the management of VAT and VAP epitomizes the diagnostic and therapeutic challenges inherent in modern intensive care. The absence of robust diagnostic criteria promotes both over- and under-diagnosis, leading to inappropriate antimicrobial use and significant clinical and ecological consequences. An effective strategy demands a comprehensive, evidence-based approach that integrates clinical evaluation, biomarker interpretation, and microbiological confirmation, ensuring that patients receive timely, targeted, and proportionate treatment—neither excessive nor insufficient.

## 6. Conclusions

TBI is one of the most complex conditions treated in intensive care units. Its interaction with the respiratory system is a prime example of the intricate interdependence of the brain–lung–immune axis. Although the physiological interaction between these systems is essential for homeostasis, it becomes a double-edged sword after acute neurological injury, predisposing patients to a wide spectrum of pulmonary complications, notably VAP and VAT.

The underlying mechanisms are multifactorial. Following severe TBI, for example, sympathetic hyperactivation, neurogenic inflammation, immune dysregulation and loss of airway reflexes can collectively weaken the host’s defenses. These processes are exacerbated by mechanical ventilation, sedation and the presence of invasive devices, all of which facilitate microaspiration and biofilm formation in the lower respiratory tract. In this context, the triple hit hypothesis, which encompasses neurogenic, iatrogenic and microbial-immunological insults, provides a useful conceptual model for understanding how brain injury progresses to systemic and pulmonary dysfunction.

From a diagnostic standpoint, distinguishing between colonization, VAT and VAP remains a persistent challenge. Conventional clinical, radiological and microbiological criteria are not specific enough, particularly in neurocritical patients, who often exhibit systemic inflammatory responses that are not infection-related. This diagnostic uncertainty can lead to inconsistent incidence rates, delayed or inadequate treatment, and excessive antibiotic exposure, exacerbating the problem of antimicrobial resistance.

Therapeutically, it is important to strike a balance between ensuring adequate and timely empirical coverage and avoiding the unnecessary use of broad-spectrum antibiotics. When limited to selected cases of TAV, nebulized antibiotic therapy may offer a localized strategy with reduced systemic toxicity, although this practice is not supported by international guidelines. Meanwhile, integrating rapid molecular diagnostic platforms, biomarker-guided decision-making and local epidemiological data could transform empirical therapy into a more targeted, management-aligned intervention.

Future research should focus on validating diagnostic algorithms tailored to neurocritical care, defining optimal thresholds for initiating antimicrobial therapy and identifying patient subgroups that would benefit most from prophylactic or early targeted therapy. Ultimately, improving outcomes for patients with TBI requires a multidisciplinary approach that harmonizes the management of neurological, respiratory and infectious diseases. This is because the brain and lungs are partners in survival, requiring both protection and close monitoring.

## Figures and Tables

**Table 1:** Clinical diagnostic criteria used for the probable diagnosis of ventilator-associated pneumonia (VAP) or ventilator-associated tracheobronchitis (VAT). (Modified from Craven et al. 2011 [14].) Abbreviations: FiO_2_: inspired oxygen fraction; PaO_2_: Arterial pressure of oxygen; CT: computed tomography; BA: Bronchial aspirate; BAL: Bronchoalveolar lavage; PCR: Polymerase Chain Reaction.

Clinical Signs and Symptoms	VAP	VAT
**At least one of these** **Plus** **One of these**	Fever (>30 °C or 100.4 °F) or leukocyte count >12,000/mm^3^ or <4000/mm^3^ New onset of purulent secretions or change in suctioning requirements	
**Oxygenation**	Worsening oxygen requirements (increasing FiO_2_) or PaO_2_/FiO_2_ ratio)	Oxygenation is generally unaffected.
**Biomarkers**	Elevated procalcitonin (>1 ng/mL)	Procalcitonin level <1 ng/mL
**Radiologic signs**		
**Chest X-Ray**	New pulmonary infiltrates or progression of pre-existing ones	No new pulmonary infiltrates or stability of pre-existing ones
**CT scan**	Consolidation or cavitation	Finding consistent with atelectasis or ARDS
**Lung ultrasound**	Dynamic air bronchograms or branched echogenic structures within the area of alveolar consolidation, with centrifugal movements with inspiration	Consolidation without dynamic air bronchograms (atelectasis)
**Microbiological criteria in quality samples (BA or BAL)**	Many polymorphonuclear leukocytes (PMNL) and many bacteria	
**Syndromic multiplex panels (PCR) (1–2 h)**	number of copies ≥ 10^6^–10^7^	number of copies ≤ 10^5^
**Microbiological culture and cut-off (48–72 h)**	Bronchoscopic BAL ≥ 10^4^ or PSB ≥ 10^3^ ufc/mL Nonbronchoscopic BAL ≥ 10^3^ ufc/mL BA ≥ 10^6^	Bronchoscopic BAL < 10^4^ or PSB < 10^3^ ufc/mL Nonbronchoscopic BAL < 10^3^ ufc/mL BA < 10^6^

**Table 2:** The measures for preventing ventilator-associated pneumonia (care bundle), as set out in the National Zero Pneumonia Plan of the Spanish Ministry of Health and international bundles.

Pneumonia “Zero” Care Bundle [46]	International Care Bundle [47,48,49,50]
1. Keep the head of the bed at an angle of more than 30 degrees, unless this is clinically contraindicated.	1. Head of bed elevation to 30° to 45°
2. Practise strict hand hygiene before and after manipulating the airway, and wear sterile, single-use gloves.	2. Hand hygiene
3. Educate and train healthcare personnel in airway management.	3. Spontaneous breathing trials
4. Promote safe extubation to reduce ventilation time.	4. Decrease sedation: daily awakening trials
5. Continuously monitor the pressure of the pneumatic seal of the tracheal tubes.	5. Cuff pressure monitoring
6. Use tracheal tubes with a continuous subglottic secretion suction system.	6. Subglottic drainage/suctioning
7. Do not routinely change the ventilator tubing.	7. Ventilator circuit manipulation
8. Administer antibiotics within 24 h of intubation to patients with decreased consciousness prior to intubation.	8. Intravenous antibiotics
9. Perform oral hygiene using a solution of 0.12–0.2% chlorhexidine.	9. Oral care/selective oral decontamination
10. Use complete selective digestive decontamination. (SDD)	10. Selective digestive (SDD) or selective oral decontamination(SOD)
--------------------------------------------------------------	11. Early mobilization
--------------------------------------------------------------	12. Gastric residual volumes
--------------------------------------------------------------	13. Probiotics

**Table 3:** Risk factors for ventilator-associated pneumonia (VAP) as reported in published studies.

Author/Year	Risk Factor	OR/HR (95% CI)
Bronchard (2004) [10]	Early VAP	
	● Aspiration before OTI	5.5 (1.9–16.4)
	● Barbiturate use	3.9 (1.2–12.8)
	● Nasal carriage MSSA	5.1 (1.9–14.0)
Lepelletier (2010) [63]	Early VAP	
	● Barbiturate use	2.6 (1.06–6.8)
	● Immunodepression	7.1 (1.6–30.7)
	● Early enteral nutrition	0.3 (0.2–0.8)
	● Early neurosurgery	0.3 (0.1–0.8)
Jovanovic (2015) [9]	Early VAP	
	● Injury of thorax	8.56 (2.05–35.7)
	● ISS	1.09 (1.03–1.15)
	● Coma	13.40 (3.12–57.6)
	Late VAP	
	● Age	1.04 (1.02–1.07)
	● ISS	1.09 (1.04–1.13)
	● Coma	3.84 (1.44–10.28)
Esnault (2017) [11]	Early	
	● Hypothermia	3.4 (1.2–10.0)
	● Thoracic AIS > 3	2.4 (1.1–5.7)
	● Culture (+) admission	4.2 (1.7–10.6)
	● Gastric aspiration	5.2 (1.7–15.9)
	● Prophylactic AB (<48 h)	0.3 (0.1–0.8)
Li * (2020) [64]	● Smoking	2.13 (1.16–3.92)
	● Tracheostomy	9.55 (3.24–28.17)
	● Blood transfusion	2.54 (1.24–5.18)
	● Barbiturate infusion	3.52 (1.68–7.40)
	● High ISS	4.65 (1.96–7.34)
	● Head AIS > 3	2.99 (1.66–5.37)
Robba (2020) [65]	● Age	0.99 (0.98–0.99)
	● Chest trauma	1.40 (1.03–1.9)
	● Prophylactic AB	0.69 (0.50–0.96)
	● H2 antagonist	2.16 (1.37–3.39)
Cáceres (2023) [66]	● Age	1.10 (1.01–1.20)
	● AIS thorax	1.42 (1.13–1.79)
	● MV on admisión	3.70 (1.24–13.5)
	● GCS < 8	2.70 (1.11–6.94)
Prieto-Alvarado * (2025) [67]	● Male	1.58 (1.23–2.02)
	● AIS > 3	2.79 (1.58–4.93)

* Meta-analysis. Abbreviations: AIS: Abbreviated Injury Scale; GCS: Glasgow coma scale; MV: mechanical ventilation; OTI: orotracheal intubation; MSSA: methicillin-sensitive *S. aureus*; ISS: Injury Severity Score.

**Table 4:** The most common microorganisms isolated in neurocritical patients with ventilator-associated pneumonia (VAP), according to different authors. Some authors differentiate between early-onset VAP (within 5–7 days of mechanical ventilation) and late-onset VAP (>7 days). (MSSA: methicillin-sensitive *S. aureus*).

		Most Frequently Isolated Microorganisms in VAP (%)			
Autor	Year/Country	1st	2nd	3rd	4rd
Bronchard [10]	2004/France				
	Early	MSSA (57.8)	*H. influenzae* (53.3)	*S. pneumoniae* (15.6)	*Enterobacteriaceae* (17.8)
Zygun [12]	2006/Canada	MSSA (31.0)	*H. influenzae* (28.0)	*E. coli* (6.0)	*Enterobacter* spp (6.0)
Agbaht [78]	2007/Spain	MSSA (34.5)	*Enterobacteriaceae* (18.8)	*H. influenzae* (17.6)	*Streptococcus* spp. (12.9)
Karvouniaris [32]	2013/Greece	*A. baumannii* (43.5)	*P. aeruginosa* (21.7)	*K. pneumoniae* (6.5)	MSSA (6.5)
Jovanovic [9]	2015/Serbian				
	Early	*A. baumannii* (47.2)	*K. pneumoniae* (15.7)	MSSA (11.7)	*P. aeruginosa* (9.8)
	Late	*A. baumannii* (51.8)	*K. pneumoniae* (17.9)	*P. aeruginosa* (16.1)	MRSA (7.1)
Esnault [11]	2017/France				
	Early	*H. influenzae* (25.0)	MSSA (24.0)	*E. coli* (11.0)	*S. pneumoniae* (7.0)
Robba [65]	2020/EU	MSSA (40.0)	*H. influenzae* (24.0)	*S. pneumoniae* (8.2)	*P. aeruginosa* (7.7)
Russo [8]	2023/Italy	MSSA (25.9)	*E. coli* (16.0)	*H. influenzae* (12.7)	*P. aeruginosa* (7.4)
Cáceres [66]	2023/Colombia	MSSA (18.0)	*K. pneumoniae* (10.0)	*E. cloacae* (8.0)	*E. coli* (7.0)
Ngxabi [79]	2024/South Africa	*K. pneumoniae* (35.0)	*A. Baumannii* (28.0)	MSSA (27)	*E. coli* (10)

## Data Availability

No new data were created or analyzed in this study. Data sharing is not applicable to this article.

## Abbreviations

The following abbreviations are used in this manuscript:

VAP	Ventilator-associated pneumonia
VAT	Ventilator-associated tracheobronchitis
IMV	Invasive mechanical ventilation
TBI	Traumatic Brain Injury
OTI	Orotracheal intubation
LRTI	Lower respiratory tract infection
ICP	Intracranial pressure
ARDS	Acute Respiratory Distress Syndrome
VILI	Ventilator-induced lung injury
CAP	Cholinergic anti-inflammatory pathway
SAS	Sympathetic-adrenomedullary system
HPA	Hypothalamic–pituitary–adrenal axis
ALI	Acute lung injury
AIS	Abbreviated injury scale
GCS	Glasgow Coma scale
ISS	Injury severity score
